# Association of Long-term Exposure to Air Pollution With Late-Life Depression in Older Adults in the US

**DOI:** 10.1001/jamanetworkopen.2022.53668

**Published:** 2023-02-10

**Authors:** Xinye Qiu, Liuhua Shi, Laura D. Kubzansky, Yaguang Wei, Edgar Castro, Haomin Li, Marc G. Weisskopf, Joel D. Schwartz

**Affiliations:** 1 Department of Environmental Health, Harvard T.H. Chan School of Public Health, Boston, Massachusetts; 2 Gangarosa Department of Environmental Health, Rollins School of Public Health, Emory University, Atlanta, Georgia; 3 Department of Social and Behavioral Sciences, Harvard T.H. Chan School of Public Health, Boston, Massachusetts; 4 Department of Epidemiology, Harvard T.H. Chan School of Public Health, Boston, Massachusetts

## Abstract

**Importance:**

Emerging evidence has suggested harmful associations of air pollutants with neurodegenerative diseases among older adults. However, little is known about outcomes regarding late-life mental disorders, such as geriatric depression.

**Objective:**

To investigate if long-term exposure to air pollution is associated with increased risk of late-life depression diagnosis among older adults in the US.

**Design, Setting, and Participants:**

This population-based longitudinal cohort study consisted of US Medicare enrollees older than 64 years. Data were obtained from the US Centers for Medicare and Medicaid Services Chronic Conditions Warehouse. The participants were continuously enrolled in the Fee-for-Service program and both Medicare Part A and Part B. After the 5-year washout period at entry, a total of 8 907 422 unique individuals were covered over the study period of 2005 to 2016, who contributed to 1 526 690 late-onset depression diagnoses. Data analyses were performed between March 2022 and November 2022.

**Exposures:**

The exposures consisted of residential long-term exposure to fine particulate matter (PM_2.5_), measured in micrograms per cubic meter; nitrogen dioxide (NO_2_), measured in parts per billion; and ozone (O_3_), measured in parts per billion.

**Main Outcomes and Measures:**

Late-life depression diagnoses were identified via information from all available Medicare claims (ie, hospital inpatient, skilled nursing facility, home health agency, hospital outpatient, and physician visits). Date of the first occurrence was obtained. Hazard ratios and percentage change in risk were estimated via stratified Cox proportional hazards models accounting for climate coexposures, neighborhood greenness, socioeconomic conditions, health care access, and urbanicity level.

**Results:**

A total of 8 907 422 Medicare enrollees were included in this study with 56.8% being female individuals and 90.2% being White individuals. The mean (SD) age at entry (after washout period) was 73.7 (4.8) years. Each 5-unit increase in long-term mean exposure to PM_2.5_, NO_2_, and O_3_ was associated with an adjusted percentage increase in depression risk of 0.91% (95% CI, 0.02%-1.81%), 0.61% (95% CI, 0.31%- 0.92%), and 2.13% (95% CI, 1.63%-2.64%), respectively, based on a tripollutant model. Effect size heterogeneity was found among subpopulations by comorbidity condition and neighborhood contextual backgrounds.

**Conclusions and Relevance:**

In this cohort study among US Medicare enrollees, harmful associations were observed between long-term exposure to air pollution and increased risk of late-life depression diagnosis.

## Key Points

**Question:**

Is long-term exposure to air pollution associated with increased risk of late-onset depression diagnosis among older adults in the US?

**Findings:**

In the US nationwide Medicare cohort with 1 526 690 late-onset depression diagnoses, statistically significant harmful associations were observed between long-term exposure to common levels of air pollution and increased risk of depression diagnosis after age 64 years, accounting for climate coexposures, neighborhood greenness, socioeconomic conditions, health care access, and urbanicity level.

**Meaning:**

This study suggests that air pollution is a potential risk factor for late-onset depression.

## Teaser

This cohort study investigates if long-term exposure to air pollution is associated with increased risk of late-life depression diagnosis among US older adults.

## Introduction

Depression in older adults is a concern and can be as important as dementia. It is estimated that in the US, the excess annual adjusted health care costs related to depression reached $27.4 million per 1 million older adults.^1^ Previous animal studies have provided critical insights into the mechanisms supporting air pollution’s association with mental illness and found that air pollutants may translocate to the central nervous system through nasal epithelial and alveolar capillary dysfunction and blood-brain barrier breakdown, thereby eliciting adverse neuroinflammatory and autoimmune responses.^2^ The various pathways involved include but are not limited to increased levels of oxidative stress in the brain, activation of the hypothalamic-pituitary-adrenal axis, and the triggering of increased release of stress hormones, which are tied to a series of cognitive and mental outcomes, including depression.^3,4^ Evidence has also shown that the process of aging can affect the immune responses in the periphery and the periphery-brain immune communication, as well as increase the levels of activated microglia leading to the production of several proinflammatory cytokines.^5^ In fact, the older adult population is observed to be at higher risk of depression related to hazardous environmental pollutants exposure due to their pulmonary and neural vulnerability to inflammatory triggers (eg, air pollutants) and have a higher chance of developing related physical comorbidities associated with worsened psychiatric health.^6^ It is possible that exposure to air pollution would first result in an increased risk of developing neurological disorders or other medical conditions in older adults as observed in current literature.^7,8^ Then, experiencing adverse neurological and physical comorbidities is further associated with the mental burden of an older adult and cumulatively leads to observed depression.^9,10^ Although depression is less prevalent among older adults as compared with the younger population, there can be serious consequences, such as cognitive impairment, comorbid physical illness, and death.^11,12^ Therefore, it is of crucial importance to study preventable risk factors for developing depression among older adults to reduce the associated health care burden.

Worldwide, studies have shown that exposure to air pollution is associated with an increased risk of adverse mental health outcomes, such as depression.^13,14,15,16^ However, potential heterogeneity in these outcomes across different subpopulations is unclear. While the evidence for acute and cross-sectional associations has increased in recent years,^17,18,19^ high-quality evidence looking at late-onset depression in long-term prospective longitudinal cohorts is lacking. To our knowledge, this study is the first to examine the associations between long-term exposure to air pollution and risk of late-onset depression incidence among the US older adult population (>64 years) in a longitudinal setting over a study period of more than 10 years.

## Methods

### Study Population

In this cohort study, we utilized the Medicare denominator file and the Chronic Conditions Warehouse (CCW), 2 privacy-protected and publicly available databases derived from the Centers for Medicare and Medicaid Services, to create an open cohort. The study population included all Medicare beneficiaries who were continuously enrolled in (1) the Medicare Fee-for-Service program and (2) both Part A (for hospital insurance) and Part B (for medical insurance) in the contiguous US from 2000 to 2016. We were able to track the enrollee’s annual residential address zip code change over the follow-up years. The year of first diagnosis for depression across all available Medicare claims was identified per unique individual and was linked with the denominator. We further applied a washout period of 5 calendar years after first enrollment, during which there were no diagnosis codes for depression. By applying the washout period and removing prevalent cases of depression in their first 5 years of follow-up, we would have a much higher chance of approximating the incidence of depression in late life. We considered that 5 years was a reasonable period to largely increase the chance that a participant was not an existing depression patient prior to the Medicare diagnosis. In the final cohort, all individuals entered the cohort on the first date of the year following the washout period and were followed up until the first diagnosis of depression across the claims, death, or end of the follow-up period. To avoid immortal time bias,^20^ we excluded the cleaning period from follow-up time. Hence, the earliest year follow-up started was 2005. This study was conducted with approval from the institutional review boards of Emory University and the Harvard T.H. Chan School of Public Health. Informed consent was waived because we conducted secondary analyses of deidentified data. We followed the Strengthening the Reporting of Observational Studies in Epidemiology (STROBE) reporting guideline.

### Exposure Assessment

We treated 3 criteria air pollutants as our exposures (1) fine particulate matter, PM_2.5_, (2) nitrogen dioxide, NO_2_, and (3) ozone, O_3_. Ambient daily pollutants predictions (24-hour mean PM_2.5,_ 1-hour maximum NO_2_, and 8-hour maximum O_3_) at 1 km×1 km–high resolution across the contiguous US from 2000 to 2016 were obtained in high-performance air pollution prediction models.^21,22,23^ The daily grid-cell level exposures were aggregated into each zip code, averaged across each year, and linked into each enrollee by zip code of residence and follow-up year. We calculated the current and 5-year–before window moving means of air pollutants for each follow-up year and each individual and treated them as the long-term exposure window.

### Outcome Identification

Depression was identified via an algorithm in CCW that combines information from all available Medicare claims (such as hospital inpatient, skilled nursing facility, home health agency, hospital outpatient, and carrier claims [including physician visits] with valid *International Classification of Diseases, Ninth Revision* or *International Statistical Classification of Diseases and Related Health Problems, Tenth Revision [ICD-9/10]* codes).^24^ We obtained the date of the first occurrence with depression diagnosis code from the CCW after the beneficiary enrolled in the Medicare Fee-for-Service program and both Part A and Part B. The specific ICD codes and algorithm for diagnosis of depression condition in the Medicare CCW are shown in eTable 1 in Supplement 1.

### Covariates Measures

The Medicare denominator file provides information on several individual-level variables, such as age at entry, age at follow-up, sex, race, Medicaid eligibility, date of death, and others. Common comorbidity conditions were also available in the Medicare CCW, such as Alzheimer disease, dementia, congestive heart failure (CHF), hypertension, stroke, chronic obstructive pulmonary disease (COPD), diabetes, and cancer (prostate, breast, colorectal, lung, endometrial). We considered them as potential modifiers of the association between air pollution and risk of depression. These medical comorbidities were considered as potential modifiers but not confounders in this study because ambient air pollution exposure is more likely a potential risk factor (cause) for them but not a downstream consequence. Further explanations have been provided in the eMethods in Supplement 1. A directed acyclic graph causal diagram showing the pathways from air pollution exposure to late-life depression and their associations with comorbidities can be found in eFigure 1 in Supplement 1. For each enrollee, based on their zip code of residence and follow-up year, we linked information on US geographical regions (northeast, southeast, southwest, west, middle west); area-level annual average meteorology on temperature and precipitation; greenness level (via Normalized Difference Vegetation Index); and neighborhood contextual backgrounds and socioeconomic status factors, such as population density, percentage below the poverty line, percentage high school education level or less, percentage Black individuals, percentage Asian individuals, percentage Hispanic individuals, smoking rate, and percentage renting. In addition, distance to the nearest hospital and percentage ambulatory visit level were controlled as proxy variables for access to medical care. We also adjusted for the Index of Concentration at the Extremes as an indicator for area-level psychosocial stress level induced by income disparity within a community.^25,26^ Details of variable sources can be seen in the eMethods in Supplement 1.

### Statistical Analysis

We applied stratified Cox proportional hazards models with a generalized estimating equation by including a cluster variable for each zip code^27^ to estimate the hazard ratios (HRs) of incident depression risk among the Medicare enrollees when exposed to long-term average air pollution (moving mean of current and past-5-years exposure). We fit single-pollutant, bipollutant, and tripollutant models to obtain the HR and its 95% CIs per 5-unit increases in long-term mean pollutant levels prior to the first diagnosis of depression and further computed the percentage (%) change in the risk based on the HRs (percentage change, % = (HR−1) × 100%). Absolute risk differences of extra depression diagnoses at the examined annual mean air pollution levels were additionally estimated for the main analysis via the formula of α × [(HR−1)/ HR]. Here, HR denotes the hazard ratio per mean exposure level increase, and α denotes the baseline depression diagnosis risk in the study population, which is the baseline average number of depression diagnoses recorded per year over the study period. The application of a generalized estimating equation model was to adjust for residual autocorrelation within zip codes by adopting robust standard errors. We stratified all models by 4 individual-level characteristics, including sex, race (Black individuals, White individuals, and other races and ethnicities [including Asian, Hispanic, Native American, Pacific Islander, and multiracial individuals]), 5-year age groups at entry, and Medicaid eligibility, to incorporate the flexible strata-specific baseline hazard functions. Race and ethnicity were assessed as variables in this study because they have been associated with a likelihood of developing depression. Also, racial and ethnic minority groups are disproportionately affected by air pollution. Race and ethnicity were categorized into 3 groups because the Centers for Medicare & Medicaid Services sort their data this way. In addition, if race and ethnicity were grouped into more detailed categories, the power of analysis for smaller groups would also be much smaller. To control for potential confounding, we adjusted for copollutants, meteorology factors (temperature and precipitation), greenness (Normalized Difference Vegetation Index), population density (as a proxy for urbanicity), neighborhood poverty composition, education level, distance to nearest hospital and percentage of persons aged 65 years and older with an annual ambulatory visit (as proxies for access to health care), percentage Black individuals, percentage Asian individuals, percentage Hispanic individuals, smoking rate, percentage renting, and Index of Concentration at the Extremes (as a proxy for community psychosocial stress). Potential residual temporal and spatial trends were controlled by including a linear term for calendar years and a factor variable for geographical regions. We additionally examined the air pollution and depression risk concentration (C) with response (R) curves via fitting natural splines of 3 degrees of freedom (DF) for each air pollutant adjusting for covariates in tripollutant models. Lower and upper extreme exposure values (<5% and >95%) were excluded when generating the curves because they are poorly constrained in spline curves. Histograms were generated along with the curves for each pollutant to show the exposure data distributions. Further details of the statistical models fitted for subanalyses can be found in the eMethods in Supplement 1. All the analyses were conducted on the Rollins High Performance Computing cluster at Emory University between March 2022 and November 2022. Analyses were conducted using R statistical software, version 4.0.2 (R Project for Statistical Computing). A 2-sided *P* < .05 was considered statistically significant.

## Results

### Study Population Characteristics

After applying the study population restriction criteria, we observed a total of 8 907 422 unique persons in the Medicare cohort, who contributed to 1 526 690 late-life depression incident diagnoses. Among them, 5 063 769 (56.8%) were female individuals. The mean (SD) age at entry was 73.7 (4.8) years. The majority of the cohort were White individuals (n = 8 031 613, 90.2%). Approximately 6.3% of the enrollees were also eligible for Medicaid coverage (medical insurance in the US for people who are socioeconomically disadvantaged). Approximately 25.2% of the total participants had CHF, and approximately 23.6% of them had COPD. The total person-years covered after the cleaning period were 59 021 872 years.^28^ Details on other demographic measures, prevalence of comorbidities, and environmental factors were summarized in Table 1.

**Table 1.** Descriptive Statistics for the Study Population After Washout Period

Characteristics	Depression cohort
No. of events	1 526 690
Population size, No.	8 907 422
Total person-years, No.	5 9021 872
Follow-up, median, y, No.	4
Age at entry, mean (SD), y	73.7 (4.8)
Sex, No. (%)	
Female	5 063 769 (56.8)
Male	3 843 653 (43.2)
Race and ethnicity, No. (%)	
Black	505 225 (5.7)
White	8 031 613 (90.2)
Other^a^	370 584 (4.2)
Medicaid eligibility, No. (%)	
No	8 346 974 (93.7)
Yes	560 448 (6.3)
Comorbidity, No. (%)	
Alzheimer disease	430 714 (4.8)
Cancer^b^	1 501 425 (16.9)
Congestive heart failure	2 248 288 (25.2)
COPD	2 103 230 (23.6)
Dementia	1 100 975 (12.4)
Diabetes	3 066 768 (34.4)
Hypertension	7 394 940 (83.0)
Stroke	1 336 408 (15.0)
Air pollutants^c^	
PM_2.5_, μg/m^3^, mean (SD)	9.6 (2.7)
NO_2_, ppb, median (IQR)	15.4 (10.4-23.1)
O_3_, ppb, mean (SD)	39.1 (4.0)
Other environmental factors^c^	
Temperature, °C, mean (SD)	14.2 (4.5)
Accumulation precipitation, median (IQR), m	1.1 (0.8-1.3)
NDVI, median (IQR)	0.3 (0.3-0.3)
Population density, people per mile^2^, median (IQR)	244.3 (46.2-970.1)
Poverty, %, median (IQR)	0.1 (0.1-0.2)
Hispanic individuals, %, median (IQR)	0.0 (0.0-0.1)
Smoke rate, %, mean (SD)	0.5 (0.1)
Income extreme index, mean (SD)^d^	0.0 (0.2)

Abbreviations: C, Celsius; COPD, chronic obstructive pulmonary disease; NDVI, Normalized Difference Vegetation Index; NO_2_, nitrogen dioxide; O_3_, ozone; PM_2.5_, fine particulate matter; ppb, parts per billion.

^a^ Other indicates Asian, Hispanic, Native American, Pacific Islander, and multiracial individuals.

^b^ Site-specific cancer (prostate, breast, colorectal, lung, endometrial).

^c^ Annual mean levels.

^d^ Community psychosocial stress proxy.

### Air Pollution Distributions

Participants were exposed to an annual mean (SD) PM_2.5_ level of 9.6 (2.7) μg/m^3^, O_3_ level of 39.1 (4.0) parts per billion (ppb), and an annual median (IQR) NO_2_ level of 15.4 (10.4-23.1) ppb at entry. We also presented the nationwide air pollution distribution maps for the 3 pollutants in 2010 (eFigure 2 in Supplement 1).

### Association of Air Pollution With Depression

All 3 pollutants were associated with an increased risk of developing depression after 64 years of age (Table 2). The HRs were slightly attenuated toward the null in bipollutant and tripollutant models. Overall, each 5-unit increase in long-term mean air pollution exposure was associated with a statistically significant percentage increase of 0.91% (95% CI, 0.02%-1.81%), 0.61% (95% CI, 0.31%-0.92%) and 2.13% (95% CI, 1.63%-2.64%) on (hazard) risk of depression for PM_2.5_, NO_2_, and O_3_ based on the tripollutant model, respectively. The effect sizes were substantially higher in low-exposure areas for PM_2.5_ and O_3_ but stayed almost the same for NO_2_. In addition, counting PM_2.5_, NO_2_, and O_3_ together, the attributable risk difference (additional depression diagnoses that would occur), if the study population was all exposed to the examined mean air pollutants levels over the study period, is 24 266 (95% CI, 16 532-31 788) cases per year based on the main tripollutant model estimation. The exposure concentration and response curves showed that the risk of depression followed approximately linearly increasing associations with all 3 pollutants in the lower exposure ranges (Figure). The curves presented additional information on how the HR varies across the exposure ranges for the 3 pollutants.

**Table 2.** Hazard Ratio (HR) and Percentage Change in Depression Diagnosis Risk and 95% CIs Among Medicare Participants per 5-Unit Increase in Long-term Mean Pollutant Exposure, 2005-2016

Model	PM_2.5_, μg/m^3^		NO_2_, ppb		O_3_, ppb	
	HR (95% CI)^a^	Change, % (95% CI)^b^	HR (95% CI)^a^	Change, % (95% CI)^b^	HR (95% CI)^a^	Change, % (95% CI)^b^
Single	1.021 (1.013-1.030)	2.13 (1.31-2.96)	1.008 (1.005-1.011)	0.79 (0.50-1.08)	1.023 (1.018-1.028)	2.26 (1.76-2.76)
PM_2.5_ plus O_3_	1.016 (1.008-1.025)	1.64 (0.82-2.48)	NA	NA	1.021 (1.016-1.026)	2.12 (1.61-2.62)
PM_2.5_ plus NO_2_	1.014 (1.005-1.023)	1.39 (0.50-2.28)	1.006 (1.003-1.009)	0.62 (0.31-0.93)	NA	NA
O_3_ plus NO_2_	NA	NA	1.007 (1.004-1.010)	0.72 (0.44-1.00)	1.022 (1.017-1.027)	2.20 (1.70-2.71)
Tripollutant	1.009 (1.000-1.018)	0.91 (0.02-1.81)	1.006 (1.003-1.009)	0.61 (0.31-0.92)	1.021 (1.016-1.026)	2.13 (1.63-2.64)
Low-level^c^	1.072 (1.055-1.090)	7.22 (5.46-9.01)	1.006 (1.003-1.009)	0.62 (0.30-0.93)	1.047 (1.038-1.057)	4.74 (3.78-5.71)

Abbreviations: NA, not applicable; NO_2_, nitrogen dioxide; O_3_, ozone; PM_2.5_, fine particulate matter; ppb, parts per billion.

^a^ Hazard ratio and 95% CIs of depression diagnosis risk per 5 unit-increase in long-term mean exposures (moving mean of current and past 5 years of exposure) adjusting for coexposures, calendar year, region, temperature, precipitation, Normalized Difference Vegetation Index, population density, community poverty, low education rate, nearest hospital distance, percentage ambulatory visit level, percentage Black individuals, percentage Asian individuals, percentage Hispanic individuals, community smoking rate, percentage renting, and income extreme index (community psychosocial stress proxy).

^b^ Percentage (%) change and 95% CIs in the depression diagnosis risk per 5-unit increase in long-term mean exposures (moving mean of current and past 5 years of exposure) adjusting for same covariates.

^c^ Tripollutant model estimates restricting to low exposure areas where annual mean exposures (2005-2016) were always lower than or equal to the current US Environmental Protection Agency long-term ambient standards of PM_2.5_ (12 μg/m^3^), NO_2_ (53 parts per billion), and World Health Organization long-term ambient standard of O_3_ (interim target 1) 50 parts per billion, respectively.

**Figure.**
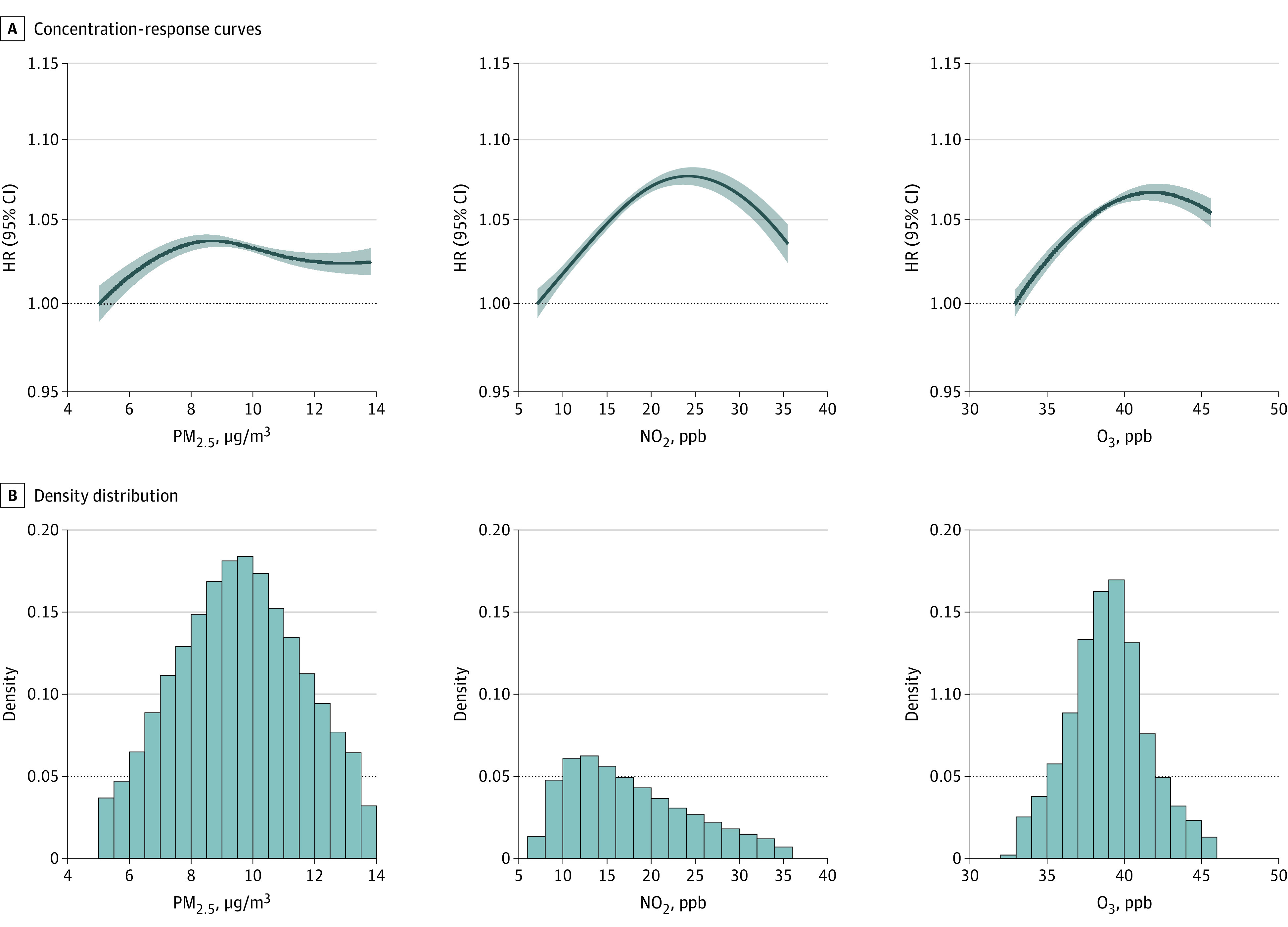
Concentration-Response Curves With Density Distributions of Pollutants The curves were derived based on a tripollutant model and are shown for the concentration ranges between the 5th to 95th percentiles of the pollutants, with the lower and upper poorly constrained extreme values excluded. HR indicates the hazard ratio with reference to the minimal exposure value. NO_2_ indicates nitrogen dioxide; O_3_, ozone; PM_2.5_, fine particulate matter; ppb, parts per billion.

### Potential Modifiers of Association Between Pollutants and Depression

We found heterogeneous strengths of associations between the 3 pollutants and depression risk in different subpopulations by demographic and community factors (eFigure 3 in Supplement 1). Results consistently showed that negative outcomes among people who were Medicaid eligible (socioeconomically disadvantaged) were likely to be associated with PM_2.5_ and NO_2_. Detailed HR estimates can be seen in Table 3. In addition, we observed modification of associations across subpopulations with or without certain comorbidities. We consistently found that older adults with comorbidities had a higher risk of developing late-life depression when exposed to the same 5-ppb increase in long-term NO_2_ exposure as compared with those without. The heterogeneity test reached statistical significance for all examined comorbidities except cancer. For PM_2.5_, only hypertension statistically significantly modified the association between PM_2.5_ and risk of depression (heterogeneity test *P* = .001). However, for O_3_, we observed effect size heterogeneity by comorbidity in the opposite direction for some conditions, including diabetes, stroke, hypertension, and CHF. Detailed estimates were shown in Table 4.

**Table 3.** Subgroup Estimates of Hazard Ratios and 95% CIs by Individual Demographic and Community Contextual Modifiers per 5-Unit Increase in Mean of Long-term Pollutants

		PM_2.5_		NO_2_		O_3_	
Modifier	Group	HR (95% CI)^a^	*P* value^b^	HR (95% CI)^a^	*P* value^b^	HR (95% CI)^a^	*P* value^b^
Sex	Male	1.016 (1.005-1.027)	.01	1.018 (1.014-1.021)	<.001	0.999 (0.993-1.005)	<.001
	Female	1.006 (0.997-1.015)		1.001 (0.997-1.006)		1.032 (1.027-1.038)	
Race	White	1.013 (1.004-1.022)	1 [Reference]	1.006 (1.003-1.009)	1 [Reference]	1.019 (1.014-1.024)	1 [Reference]
	Black	1.037 (1.010-1.064)	.08	1.035 (1.028-1.042)	<.001	0.983 (0.967-1.000)	<.001
	Other^c^	0.926 (0.906-0.947)	<.001	0.972 (0.965-0.979)	<.001	1.073 (1.058-1.088)	<.001
Medicaid eligibility	No	1.002 (0.993-1.011)	<.001	1.003 (1.000-1.006)	<.001	1.021 (1.016-1.026)	.72
	Yes	1.060 (1.041-1.079)		1.030 (1.025-1.035)		1.023 (1.013-1.034)	
Population density	Quartile 1	1.039 (1.027-1.052)	1 [Reference]	1.032 (1.025-1.039)	1 [Reference]	1.026 (1.016-1.036)	1 [Reference]
	Quartile 2	0.992 (0.978-1.007)	.28	1.029 (1.023-1.036)	.55	1.014 (1.004-1.023)	.06
	Quartile 3	0.972 (0.957-0.988)	<.001	1.000 (0.995-1.006)	<.001	1.026 (1.018-1.035)	.93
	Quartile 4	0.907 (0.892-0.922)	<.001	0.977 (0.972-0.981)	<.001	1.035 (1.027-1.043)	.16
Proportion of Hispanic individuals living in an area	High	0.985 (0.975-0.996)	<.001	0.998 (0.995-1.001)	<.001	1.025 (1.019-1.031)	.01
	Low	1.034 (1.023-1.045)		1.022 (1.018-1.027)		1.012 (1.003-1.021)	
Area poverty	Less poor	1.009 (1.000-1.019)	.92	1.006 (1.003-1.009)	.26	1.026 (1.020-1.031)	<.001
	Poor	1.009 (0.993-1.024)		1.009 (1.004-1.014)		0.998 (0.988-1.008)	
Income extreme index^d^	Quintile 1	1.032 (1.018-1.046)	1 [Reference]	1.013 (1.008-1.019)	1 [Reference]	0.993 (0.982-1.003)	1 [Reference]
	Quintile 2	1.033 (1.019-1.047)	.93	1.015 (1.010-1.020)	.70	1.017 (1.007-1.028)	.001
	Quintile 3	1.013 (0.999-1.026)	.03	1.009 (1.004-1.014)	.14	1.022 (1.013-1.032)	<.001
	Quintile 4	1.007 (0.993-1.021)	.01	1.007 (1.002-1.011)	.03	1.030 (1.021-1.039)	<.001
	Quintile 5	0.953 (0.938-0.968)	<.001	0.993 (0.988-0.997)	<.001	1.034 (1.025-1.043)	<.001

Abbreviations: HR, hazard ratio; NO_2_, nitrogen dioxide; O_3_, ozone; PM_2.5_, fine particulate matter.

^a^ Hazard ratio per 5-unit increase in annual mean exposure (moving means of current and past 5 years of exposure) adjusting for selected covariates in different subgroups.

^b^ Interaction term *P* value at different levels.

^c^ Other indicates Asian, Hispanic, Native American, Pacific Islander, and multiracial individuals.

^d^ Community psychosocial stress proxy.

**Table 4.** Subgroup Results by Comorbidities Condition Showing Hazard Ratio (HR) in Depression Diagnosis Risk and Its 95% CIs per 5-Unit Increase in Mean of Long-term Pollutant Exposure

Subpopulation	PM_2.5_, μg/m^3^		NO_2_, ppb		O_3_, ppb	
	HR (95% CI)^a^	*P* value^b^	HR (95% CI)^a^	*P* value^b^	HR (95% CI)^a^	*P* value^b^
With AD	1.021 (1.002-1.040)	.16	1.027 (1.020-1.033)	<.001	1.039 (1.028-1.050)	.002
Without AD	1.006 (0.997-1.015)		1.004 (1.001-1.007)		1.020 (1.015-1.025)	
With cancer^c^	1.024 (1.009-1.039)	.05	1.009 (1.004-1.014)	.31	1.016 (1.007-1.024)	.21
Without cancer	1.006 (0.997-1.016)		1.006 (1.003-1.009)		1.022 (1.017-1.027)	
With CHF	1.006 (0.994-1.019)	.78	1.018 (1.014-1.022)	<.001	1.014 (1.008-1.021)	.03
Without CHF	1.008 (0.999-1.018)		1.000 (0.997-1.003)		1.024 (1.018-1.029)	
With COPD	1.010 (0.998-1.022)	.80	1.013(1.009-1.018)	<.001	1.019 (1.013-1.026)	.66
Without COPD	1.008 (0.999-1.018)		1.002 (0.998-1.005)		1.021 (1.016-1.027)	
With dementia	1.014 (1.000-1.028)	.28	1.025 (1.021-1.030)	<.001	1.020 (1.012-1.027)	.72
Without dementia	1.005 (0.995-1.014)		1.000 (0.997-1.003)		1.021 (1.016-1.027)	
With diabetes	1.007 (0.995-1.018)	.46	1.011 (1.007-1.015)	<.001	1.012 (1.005-1.019)	.003
Without diabetes	1.013 (1.003-1.023)		1.002 (0.999-1.005)		1.025 (1.020-1.031)	
With HRT	1.012 (1.003-1.021)	.001	1.008 (1.005-1.011)	<.001	1.017 (1.012-1.023)	<.001
Without HRT	0.977 (0.961-0.994)		0.985 (0.980-0.990)		1.047 (1.037-1.057)	
With stroke	1.017 (1.004-1.031)	.19	1.012 (1.007-1.016)	.008	1.014 (1.006-1.021)	.05
Without stroke	1.006 (0.997-1.015)		1.004 (1.001-1.007)		1.023 (1.018-1.028)	

Abbreviations: AD, Alzheimer disease; CHF, congestive heart failure; COPD, chronic obstructive pulmonary disease; HRT, hypertension; NO_2_, nitrogen dioxide; O_3_, ozone; PM_2.5_, fine particulate matter; ppb, parts per billion.

^a^ Hazard ratio and 95% CIs of depression diagnosis risk per 5 units increase in long-term mean exposures (moving mean of current and past 5 years of exposure) adjusting for coexposures, calendar year, region, temperature, precipitation, Normalized Difference Vegetation Index, population density, community poverty, low education rate, nearest hospital distance, percentage ambulatory visit level, percentage Black individuals, percentage Asian individuals, percentage Hispanic individuals, community smoking rate, percentage renting, and income extreme index (community psychosocial stress proxy).

^b^ *P* for difference based on heterogeneity test.

^c^ Site-specific cancer (prostate, breast, colorectal, lung, endometrial).

### Subanalyses

In our subanalyses, we observed harmful associations between air pollution exposure and late-life depression over varied lag windows. In particular, the associations for PM_2.5_ were statistically significant over lag0 (current year) − lag2 (2 years prior) with reduced effect size for prior years while the associations for NO_2_ and O_3_ remained statistically significant across all exposure windows (lag 0 − lag5) (eTable 2 in Supplement 1). Second, we computed E-values for each of the pollutant-specific main estimates to indicate the possible residual confounding strength (eTable 3 in Supplement 1). In addition, crude model results without adjusting for any covariates besides the air pollutants indicated strong potential of confounding by the covariates we controlled for, especially for NO_2_. Related estimates can be seen in eTable 4 in Supplement 1. The annual mean (SD) of residential air pollution exposure among participants with vs without comorbidities over the study period was presented in eTable 5 in Supplement 1. The distributions of residential ambient air pollutant level differences before and after moving into a new address among participants with comorbidities and who moved over the study period were presented in eFigure 4 in Supplement 1.

## Discussion

In this nationwide Medicare cohort study, we found that long-term residential ambient exposure to PM_2.5_, O_3_, and NO_2_ was associated with increased risk of depression diagnosis in older adults. These findings were robust to single, bipollutant, or tripollutant model settings, as well as restricted to areas where the air pollution levels were always below current long-term regulation standards over the study period. The subgroup results showed effect size heterogeneity across different subpopulations with consistent findings showing greater associations with PM_2.5_ and NO_2_ among socioeconomically disadvantaged groups (Medicaid eligible) in terms of late-life depression risk. In addition, older adults with cardiovascular disease, respiratory, metabolic, or neurological comorbidities were more sensitive to NO_2_.

In a recent systematic review, the authors included a total of 39 epidemiological studies on air pollution exposure and depression published until May 2021. Based on the meta-analysis, they found an increased risk of depression for a 1 μg/m^3^ increase in long-term PM_2.5_ exposure (relative risk [RR], 1.074; 95% CI, 1.021-1.129), and a 1 ppb increase in long-term NO_2_ exposure (RR, 1.037, 95% CI, 1.011-1.064).^18^ The evidence with PM_2.5_ is the strongest with less evident heterogeneity.^29^ Here, we observed that long-term exposure to increased levels of O_3_ was associated with the highest risk increase in late-life depression diagnosis in the Medicare group as compared with PM_2.5_ and NO_2_, adjusting for environmental coexposures, population density, and several neighborhood factors. This finding with O_3_, if validated to be true, informs us of the importance of long-term ambient O_3_ regulation because it is one of the key pollutants projected to increase for certain regions under future climate change scenarios.^30^ Although the current evidence on the association between air pollution and depression rises increasingly, this nationwide Medicare study based on a longitudinal setting provides unique and quantitative knowledge on the risk of late-onset depression incidence from long-term residential exposure to criteria pollutants among older adults in the US. The observed strengths of associations in this study are smaller than the current pooled meta-analysis findings. Yet, these study findings were specifically targeting late-life depression diagnosis risk and were based on a more comprehensive covariates adjustment.

More studies have found that exposure to atmospheric particulate matter (PM) at commonly occurring levels leads to both acute and chronic inflammation in the brain. Researchers from Mexico showed that air pollution in Mexico City was associated with central nervous system inflammatory and neurodegenerative changes in both humans and animals.^31^ Besides neuroinflammation, increased oxidative stress is another possible pathway. It has been found that inhaled PM can activate early markers of oxidative stress in a rat model.^32^ Particle-exposed mice were observed to show increased depressive symptoms and decreased spatial learning and memory in another study conducted in Columbus, Ohio.^33^ In humans, studies have suggested that long-term exposure to increased levels of atmospheric particles is linked with altered brain structure, smaller total cerebral brain volume, reduced white matter, as well as leaking capillaries and extravascular lipids in the white matter using magnetic resonance imaging techniques, all of which may act as negative triggers for developing psychiatric illness.^34,35,36^ Nitrogen dioxide is a well-known outdoor pollutant mainly from traffic emissions. Evidence has indicated that, like PM, NO_2_ also promotes neuroinflammation. Recently, in a mouse model, anxiety-like and depression-like behaviors were found in male mice after NO_2_ exposure for 4 weeks but not in female mice.^37^ In the same study, authors found that the inhalation of NO_2_ caused damage to the ultrastructure of myelin sheath and caused the abnormal expression of mental illness–related genes in male mice. Interestingly, the sex difference (greater associations among male individuals) observed for the association between this particular pollutant and risk of depression in our study echoed with this study. In an in vivo screening study to determine the neurological hazards of NO_2_ using Wistar rats, researchers found that NO_2_ also induced oxidative stress in the brain.^38^ It is also possible that the harmful associations we observed with NO_2_ are attributed to other highly correlated air pollutants with similar emission sources, such as polycyclic aromatic hydrocarbons (PAHs), which were previously found to be associated with neurotoxicity.^39,40,41^ On the other hand, although the evidence is much less established, ambient ozone exposure has also been observed to be adversely associated with an increased risk of mental illness. Researchers found that chronic ozone inhalation was associated with memory impairment and anxiety-like and depression-like outcomes in a rodent translational model of neurobiological oxidative stress.^42^ Neurobiological studies suggested that ozone reduces dopaminergic neurons, increases lipid peroxidation,^43^ and causes changes in microglial activation and cell morphology in the substantia nigra and striatum.^44^

Socioeconomically disadvantaged individuals were observed to be at a much higher risk of late-life depression in this study. They are simultaneously exposed to both social stress and poor environmental conditions, including air pollution.^45^ An integrated and multilevel model has been proposed to describe how exposure to severe stress resulting from poor socioeconomic conditions and air pollution exposure simultaneously can activate several neuroimmunologic pathways leading to a higher risk of inflammation induced by acute environmental or personal triggers among them as compared with the general population.^46^ Additional discussion on other subanalyses findings is shown in the eDiscussion in Supplement 1.

In this study, we also looked at comorbidities as modifiers of the association between air pollution exposure and the risk of developing late-onset depression, which, to our knowledge, has rarely been looked into. We found that, especially for NO_2_ (and with certain comorbidities for PM_2.5_), those with comorbidities were associated with a higher risk of developing late-life depression. It indicates that there can be mental as well as physical health benefits for older adults with morbidities from air pollution regulation. Individuals with comorbidities are observed to have a less healthy lifestyle, weaker social engagement, and a higher chance of complex medication intake, all of which can predispose the person to a more susceptible nervous system with hyperactivation of certain preinflammatory factors (ie, cytokines, microglia) that are sensitive to neuroinflammation triggers, including air pollution exposure.^47^ However, we observed inconsistent findings with O_3_. The discrepant findings for different pollutants may be related to the negative correlation between NO_2_ and O_3_, as well as different biological mechanisms.

This study is novel in that, to our knowledge, it is the first nationwide study on long-term exposure to air pollution and risk of late-life depression diagnosis after 64 years of age among US Medicare enrollees with comprehensive adjustment of covariates, including climate coexposures (temperature, precipitation), greenness, neighborhood socioeconomic status, health care access, urbanicity, and others. First, we covered a total of 8 907 422 unique persons in the Medicare cohort, who contributed to 1 526 690 late-life depression incidence diagnoses (approximately 1.5 million cases) and 59 021 872 years of total person-years. Second, with the application of a 5-year washout period, we increased the chance of capturing the first diagnosis of late-life depression cases, rather than prevalent cases. Third, the application of nationwide high-resolution spatial-temporal air pollution predictions enabled us to assign air pollution exposure based on residential zip code area for each enrollee as well as to cover residents in both city and rural areas where air pollution monitoring is lacking. Last, the modification analysis uncovered a list of individual-level and community-level modifiers, such as comorbid conditions, sex, race, and socioeconomic conditions.

### Limitations

This study also has several limitations. First, outcome misclassification was likely because we used Medicare to create the nationwide cohort, which is an administrative health care database that relies on primary diagnosis ICD codes for defining a case of depression. However, the application of a 5-year washout period at entry as well as the CCW identification algorithm that combines information from all available Medicare claims (ie, hospital inpatient, skilled nursing facility, home health agency, hospital outpatient, and physician visits) reduced the measurement error. Second, unmeasured confounding was likely from the lack of control for potential individual-level risk factors of depression. However, we used ambient air pollution exposure (not personal air pollution exposure) as the exposure, and ambient air pollution is less correlated with individual risk factors but more with area-level factors we already controlled for.^48^ Third, residual exposure measurement error was also likely in that we linked in the exposures based on the residential zip code area instead of the specific address and did not have information on other physical locations of the participants besides their home addresses.

## Conclusions

In this cohort study among US nationwide Medicare enrollees over the study period, we observed statistically significant harmful associations between long-term exposure to elevated levels of air pollution and increased risk of late-life depression diagnosis. The study findings have implications for both environmental regulation and public health management. We hope this study can inspire researchers to further consider possible environmental risk factors (such as air pollution and living environment) for the prevention of geriatric depression, to understand the disease better moving forward, and to improve the delivery of mental health care services among older adults. Due to the high prevalence and universal exposure to the ambient environment, if statistically significant associations could be established for modifiable risk factors of depression, such as air pollution, preventive population-based solutions could be applied to help control the disease burden through air quality regulation, emission control, and greener planning for living environments. In addition, the effect size heterogeneity findings in this study showed that there can be differences in the magnitude of the associations between air pollutants and risk of depression depending on both participant-specific characteristics and community contextual characteristics. However, the mechanisms for why we are seeing these health disparities remain unclear and are worth further exploration. Therefore, the regulation and health care efforts would also need to take into account the disparities underlying.

In the next steps, researchers are encouraged to apply more advanced modern techniques (such as epigenetic analyses and functional magnetic resonance imaging) to investigate the underlying biological mechanisms, to conduct better-designed population-based longitudinal studies among other cohorts with comprehensive confounding control, and to enable the possibility of exploring sensitive exposure windows and health disparities for targeted efforts. Future studies should also be designed to measure depression onset with better assessment of symptoms, especially among older patients, as well as to establish causal evidence for the observed associations using causal modeling techniques and quasi-experimental designs.

## Notes

Supplement 1. **eTable 1.** ICD diagnosis codes for depression condition in the Medicare CCW (Reference period: 1-year, revised 02/2021) **eTable 2.** Lag-specific estimates for the associations between exposure to air pollutants and risk of late-life depression diagnosis in the Medicare over current (lag0) and past 5 years (lag1 – lag5) exposure window **eTable 3.** E-values at risk ratio scale for the main estimates **eTable 4.** Hazard ratio (HR) of depression diagnosis risk and 95% CIs among the Medicare participants over 2005-2016 per 5 units increase in long-term average pollutant exposure (pollutant crude model results) **eTable 5.** Annual mean (SD) residential exposure to air pollutants among participants with comorbidities vs without over study period **eFigure 1.** Directed acyclic graph (DAG) causal diagram showing the pathways from air pollution exposure to late-life depression and their relationships with comorbidities **eFigure 2.** Spatial distribution maps of air pollutants studied. The three panels present the average concentrations of a) annual average PM_2.5_ (μg/m^3^), b) NO_2_ (ppb), and c) O_3_ (ppb) at zip code level across the contiguous United States in 2010, respectively **eFigure 3.** Sub-group results by individual and neighborhood factors **eFigure 4.** Distributions of residential ambient air pollutants level differences post - pre moving into a new address among participants with comorbidities over the study period

Supplement 2.
Data Sharing Statement
