# Supplementary material for: Association of Long-term Exposure to Air Pollution With Late-Life Depression in Older Adults in the US
Source: JAMA Netw Open. 2023 Feb 10;6(2):e2253668. doi: 10.1001/jamanetworkopen.2022.53668 (PMC9918878; doi:10.1001/jamanetworkopen.2022.53668)
Supplement: Supplement 1. — eTable 1. ICD diagnosis codes for depression condition in the Medicare CCW (Reference period: 1-year, revised 02/2021) eTable 2. Lag-specific estimates for the associations between exposure to air pollutants and risk of late-life depression diagnosis in the Medicare over current (lag0) and past 5 years (lag1 – lag5) exposure window eTable 3. E-values at risk ratio scale for the main estimates eTable 4. Hazard ratio (HR) of depression diagnosis risk and 95% CIs among the Medicare participants over 2005-2016 per 5 units increase in long-term average pollutant exposure (pollutant crude model results) eTable 5. Annual mean (SD) residential exposure to air pollutants among participants with comorbidities vs without over study period eFigure 1. Directed acyclic graph (DAG) causal diagram showing the pathways from air pollution exposure to late-life depression and their relationships with comorbidities eFigure 2. Spatial distribution maps of air pollutants studied. The three panels present the average concentrations of a) annual average PM2.5 (μg/m3), b) NO2 (ppb), and c) O3 (ppb) at zip code level across the contiguous United States in 2010, respectively eFigure 3. Sub-group results by individual and neighborhood factors eFigure 4. Distributions of residential ambient air pollutants level differences post - pre moving into a new address among participants with comorbidities over the study period [file jamanetwopen-e2253668-s001.pdf]

Supplemental Online Content

Qiu X, Shi L, Kubzansky LD, et al. Association of long-term exposure to air pollution with late-life depression in older adults in the US. *JAMA Netw Open.* 2023;6(2):e2253668. doi:10.1001/jamanetworkopen.2022.53668

eTable 1. ICD diagnosis codes for depression condition in the Medicare CCW (Reference period: 1-year, revised 02/2021)

eTable 2. Lag-specific estimates for the associations between exposure to air pollutants and risk of late-life depression diagnosis in the Medicare over current (lag0) and past 5 years (lag1 – lag5) exposure window

eTable 3. E-values at risk ratio scale for the main estimates

eTable 4. Hazard ratio (HR) of depression diagnosis risk and 95% CIs among the Medicare participants over 2005-2016 per 5 units increase in long-term average pollutant exposure (pollutant crude model results)

eTable 5. Annual mean (SD) residential exposure to air pollutants among participants with comorbidities vs without over study period

eFigure 1. Directed acyclic graph (DAG) causal diagram showing the pathways from air pollution exposure to late-life depression and their relationships with comorbidities

eFigure 2. Spatial distribution maps of air pollutants studied. The three panels present the average concentrations of a) annual average $PM_{2.5}$ ($\mu g/m^3$), b) NO_2 (ppb), and c) O_3 (ppb) at zip code level across the contiguous United States in 2010, respectively

eFigure 3. Sub-group results by individual and neighborhood factors

© 2023 Qiu X et al. *JAMA Network Open.*

eFigure 4. Distributions of residential ambient air pollutants level differences post - pre moving into a new address among participants with comorbidities over the study period

This supplemental material has been provided by the authors to give readers additional information about their work.

© 2023 Qiu X et al. *JAMA Network Open.*

eMethods

Exposure assessment

Using the grid cells predictions, we aggregated the exposures to each ZIP code by 1) averaging the predictions at grid cells whose centroids were inside the polygonal area for general ZIP codes, or 2) assigning the prediction at the nearest grid cell for other ZIP codes that do not have polygon representations, for example an apartment building. This aggregation is to allow us to link the exposures to the residential address of each beneficiary since ZIP code is the smallest level of geographical information offered in the Medicare data. Annual average levels were computed from the daily exposures for each year. Then, we used the annual average exposure in each ZIP code, for each calendar year, as the exposure estimates for each included Medicare beneficiary based on their ZIP code of residence at each follow-up year. Since residential address is updated annually for each beneficiary in Medicare, we were able to capture the residential history over the study period for the study population and have less exposure measurement error.

Covariates

The temperature, accumulation precipitation data were obtained from the 4km GridMET climate data predictions (Climatology Lab 2021 GridMET data.[1] The GridMET project blends spatial attributes of gridded climate data from the parameter-elevation relationships on independent slopes model data with desirable temporal attributes from regional reanalysis (NLDAS-2) to provide high- resolution (1/24th degree ~4 km) gridded datasets of surface meteorological variables in the contiguous US.[2] Normalized difference vegetation index (NDVI) was used as a proxy for greenness level and was obtained from NASA’s MODIS satellite (MOD13C2)[3] ZIP code specific geographical regions of US (northeast, southeast, southwest, west, middle west) were obtained from National Geographic Organization Unites States Region Map.[4] Population density was obtained from the Socioeconomic Data and Applications Center (SEDAC).[5] The area-level socioeconomic (SES) variables (poverty, education, percent Black, percent Asian, percent Hispanic, percent renting) were obtained from the 2000 and 2010 US census, and the American Community Surveys after 2010.[6-8] The behavioral risk factors (i.e. smoking rate) were obtained from Behavioral Risk Factor Surveillance System (BRFSS) between 2000 and 2016,[9] and healthcare access data (nearest hospital distance, percent ambulatory visit level) were obtained from the Environmental Systems Research Institute (ESRI) hospital file and the Dartmouth Health Atlas data.[10,11] The income extreme index (ICE), used as an indicator for area-level psychosocial stress level induced by income disparity within community, was obtained from Harvard’s Public Health Disparities Geocoding Project [12] and was based on the algorithm of previous publication of the Dr. Nancy Krieger’s lab.

Statistical methods

To better inform policy, we conducted a similar tri-pollutant model when restricting to low exposure areas where annual average exposures were always lower than/equal to the current US EPA long-term ambient standards of $PM_{2.5}$ (12 μg/m^3), NO_2 (53 ppb), and WHO long-term ambient standard of O_3 (interim target 1) 50 ppb over the study period.[13,14]

To assess potential effect modification (EM) by individual and neighborhood factors, we included multiplicative interaction terms between potential modifiers and each of the three pollutants. We examined potential interaction by sex (Males vs. Females), race groups (White vs. Black vs. Other races), individual Medicaid eligibility (Yes vs. No), population density (divided by quartiles, Q1 - Q4, low to high), area percent of Hispanic population (High vs. Low, divided by median percentile), area percent of individuals in poverty (Poor vs. Less poor using cutoff of over 20% residents living under poverty in the area) and area ICE (divided by quintiles Q1 - Q5, low to high). Previous studies have observed different risks of developing depression among populations with certain comorbidities, especially among older adults. [15,16] Therefore, we estimated the air pollution – depression associations among Medicare enrollees across different comorbidities groups with or without each of the following conditions in stratified models: cancer (site-specific: prostate, breast, colorectal, lung, endometrial), diabetes, COPD, stroke, AD, dementia, hypertension, and CHF.

© 2023 Qiu X et al. *JAMA Network Open.*

Medical comorbidities were examined in our study as potential modifiers on the association between long-term air pollution exposure and risk of late life depression. We have added the Directed Acyclic Graph (DAG) causal diagram (**eFigure 1**) to facilitate the understanding of the causal positions of comorbidities in this research question.

Based on the causal inference framework, ***Hernán MA, Robins JM (2020). Causal Inference: What If. Boca Raton: Chapman & Hall/CRC.*** **https://www.hsph.harvard.edu/miguel-hernan/causal-inference-book/.**[17] **A confounder** is a variable which is the common cause of both the exposure and the outcome. This means that in order for a factor to act as a valid confounder in this study, it needs to be a cause of the ambient air pollution we studied, which is not true since ambient air pollution level can only be directly influenced by either emission sources, climate conditions or other area-level SES or atmospheric factors.

A mediator is another type of variables which sits in the middle of a causal pathway from the exposure to the outcome of interest and it acts as both a downstream consequence of the exposure and an upstream cause of the final outcome (see middle pathway from air pollution (exposure) -> comorbidities (type I) -> depression (outcome). The scenario when the medical comorbidities act as risk factors for depression and air pollution increases the risk of these comorbidities falls in this type of pathway, therefore, the various medical comorbidities are actually mediators instead of confounders in this study. Importantly, adjusting for a mediator in the model would often result in a biased effect estimation towards the direct effect of exposure on outcome and possibilities for over-adjustment.[18] rather than estimating the actual total effect of the exposure on outcome that includes the pathway mediated through this mediator.

A collider is a factor that sits as a downstream consequence of both the exposure and outcome of interest. In this study, it happens when both exposure to air pollution and developing depression adds to the risk of developing the medical conditions. (type II comorbidities in **eFigure 1**). Chronic illness may increase the risk of depression or the duration of depressive episode. Or alternatively, major depression may also predispose individuals to certain chronic illnesses and act as a risk factor for these medical conditions**.**[19] In this case, those types of comorbidities act as a collider and again not a confounder. And adjusting for them would instead lead to selection bias to the final estimation instead of correcting for the residual confounding bias.[20]

Therefore, as a summary, medical comorbidities in this specific research question are not considered as confounders, but rather as mediator or possible collider for the exposure-outcome pathways. In this study, we are interested to see how having each of the comorbidities can further influence the effect of air pollution on developing depression in older adults, thus we were interested in how having these conditions may modify the main association of interest between air pollution and depression (as an effect modifier, either it belongs to the mediator or collider scenarios.)

To test the robustness of the findings, we ran a set of sensitivity and sub-analyses. First, we examined the exposure lag-specific associations with outcome risk across the long-term exposure window (from the current year: lag 0 to 5 years before: lag5). Second, to check the strength of residual confounding, we computed pollutant-specific E-values for main HR estimates as well as their 95% confidence interval. Here, E-value is defined as the minimum strength of association on the risk ratio scale that an unmeasured confounder would need to have with both the exposure and the outcome to fully explain away a specific exposure-outcome association, conditional on the measured covariates.[21] Crude models without adjusting for any covariates were ran to compare the crude estimates with covariates adjusted estimates. To assess the potential difference in ambient air pollution levels among people with comorbidities vs. without, we computed and further compared the annual average (standard deviation, SD) of residential ambient air pollutants levels among participants with comorbidities vs. without over the study period. To have a more intuitive and direct comparison in terms of the decision to move to a new place with different opportunity of air pollution exposure among those with comorbidities, we obtained those participants with any of the comorbidities and who actually moved during the study period (we have their annually updated address zip code), and further computed the differences in the local ambient air pollution levels pre- and post- moving, to see if there is any pattern showing having comorbidities can affect where the individuals chose to live and the opportunity of significantly changing their residential air pollution exposure.

© 2023 Qiu X et al. *JAMA Network Open.*

eDiscussion

Sex appeared to be a modifier for the associations between air pollution and risk of depression. In this study, we found that males were more susceptible to long-term $PM_{2.5}$ and NO_2 exposure as compared to females while females were more susceptible to long-term O_3 exposure. Higher sensitivity of males to NO_2 has been observed in previous studies.[22,23] Mice-based animal models have also revealed that ambient NO_2 exposure can induce male sex-specific impairs in myelin sheath. The lack of prolactin among males, usually an anti-inflammatory product in females, may explain part of the increased risk of mental disorders in males caused by NO_2 exposure.[24] We also observed certain racial disparity in that Black population was observed to be more affected by $PM_{2.5}$ and NO_2 as compared to white population. This finding is in line with the finding we discovered with socioeconomically disadvantaged individuals since the proportion of socioeconomically disadvantaged individuals is much higher in Black vs. White population. The Black population is more likely to have a higher risk of neuro-inflammation triggered by air pollutants in that they are disproportionally, cumulatively exposed to higher exposure levels, and may have an existing more susceptible nervous system rooting from other social, environmental stressors. However, this is not the case with O_3. The stronger air pollution – depression associations in areas with lower population density quartile may indicate stronger effect size in the lower exposure ranges (the case with more rural areas). In fact, in our C-R curves examination as well as low-level analysis, we did see stronger effect estimates for the lower vs. higher exposure levels. In addition, we observed that enrollees who lived in high Hispanics communities were less sensitive to $PM_{2.5}$ and NO_2 exposure, but not O_3. Similar findings were seen in previous short-term air pollution exposure – mental hospitalization risk in Medicare.[25] Stronger social cohesion as well as multi-generational extended home structures among Hispanic communities have been proposed to explain the higher resilience among older adults from Hispanic communities.[26,27] The effect heterogeneity we saw across communities with different ICE levels remains unclear and warrants further investigation.

We would like to note that several protective associations were found among certain sub-populations based on our sub-group analysis. For example, protective associations of air pollution – depression risk were observed among older adults of other races for $PM_{2.5}$ and NO_2, among those of black race for O_3, among individuals living in areas at higher percentiles of population density and ICE for $PM_{2.5}$ and NO_2, and among individuals living in areas with high Hispanic proportion for $PM_{2.5}$. Second, we also observed slightly negative associations between exposure to $PM_{2.5}$, NO_2 and risk of depression diagnosis among individuals without hypertension. All these protective findings may indicate potential higher residual confounding bias for those subgroups. It is likely that there could be non-linear confounding structure underlying the exposure – outcome relationship for certain continuous confounders which leads to insufficient covariates adjustment among those sub-populations where the confounder – exposure or confounder – outcome relationship does not follow linearity. In this case, more bias is likely for those sub-populations under the linear covariates adjustment approach we adopted here. It could also be due to the possibility that the air pollution exposure levels in certain sub-populations are different from the overall population and sits at the range where the outcome risk follows a true declining curve with the increase of exposure. Further studies are needed to investigate these findings and potential non-linear modeling of the covariates adjustment or more advanced effect modification modeling techniques should be considered.

In addition, we would like to note that we found slightly different effect sizes in single-, bi- and tri-pollutant settings with tri-pollutant models exhibited the modest association strength after accounting for the two co-pollutants. A multi-pollutant approach is preferred in air pollution epidemiological studies in that co-pollutants are often important confounders that can cause bias in effect estimation if unadjusted.[28] In this study, the effect estimates generated from the tri-pollutant model were adjusted for co-pollutants, therefore are preferred as our main findings.

Besides, stronger associations were observed in low-exposed areas. Our results indicated that for per 5 units increase in the ambient air pollutants level, the associated hazard risk (effect slope) increase in depression is higher and sharper (effect slope) for increases in the low-exposed ranges as compared with increases in the high-exposed ranges. It should be noted that this finding does not conflict with our main finding that increased levels of air pollution exposure is

© 2023 Qiu X et al. *JAMA Network Open.*

associated with higher risk of depression, but rather complement the main findings in that we additionally found that for low-exposure areas, the incremental increase in depression risk associated with same unit change in air pollution level in low-exposed areas is higher than in high-exposed areas. Previous studies looking at mortality and neurological disorders in U.S. older adults have also seen similar findings with bigger effect size in low air pollution settings.[29,30] This could be possibly due to that at low air pollution areas, particles tend to carry a smaller size and have bigger surface/mass ratio. Smaller particles can be more easily inhaled and penetrated into human body, and even the brain, causing systematic and neuro-inflammation in the central nervous system.[31] More studies are needed to further verify the stronger findings at low levels and to explore the underlying related mechanisms.

Furthermore, in thinking of the relationship between ambient air pollution and medical comorbidities and the possibility that having certain comorbidity conditions may affect where the individuals live and thus the opportunity of air pollution exposure, in our sensitivity analyses, we found that on average, people with comorbidities lived in areas with slightly higher (but not significant) ambient $PM_{2.5}$, NO_2 levels as compared to those without the comorbidities. However, these results only indicate that, on average, people with these comorbidities tended to live in places with slightly higher residential air pollution levels and this may well be due to that air pollution acts as a harmful risk factor for developing these comorbidities. Underlying temporality of the exposure and comorbidities is unclear. In addition, we found that for all the comorbidities, the distributions of the air pollution level pre-post moving differences are all close-to-normal distributions with the mean around 0, which means that although it is likely that having medical conditions can affect one's decision to move, however on average, air pollution level at their new address they chose to move did not differ substantially from the old address and can be either lower, higher or almost the same as the old address, and the decision to move is rather a random choice independent of the local ambient air pollution level. Movers are generally unaware of the air pollution concentrations at the locations they consider moving to. Based on the epidemiological confounding structure, it is more likely that air pollution acts as a trigger for developing these other chronic conditions and increases their risk, and having these conditions, further increases the risk of developing depression especially in older adults with the chance of reverse causation between depression and the medical conditions. Therefore, comorbidities would either act as a mediator (type I) or collider (type II) in any case and should not be controlled for as a "confounder" in the analyses.

Ambient environmental exposure, such as air pollution, has its ubiquitous feature and affects all of us. As we presented in our results, the attributable additional late-life depression diagnoses when people are exposed to the observed average air pollution level in the U.S. are not negligible. The reported effect sizes here for air pollutants, although not as strong as existing identified personal risk factors for depression, still have meaningful indication on large population-based intervention for late-life depression via stricter ambient air pollution regulation. Air pollution is preventable and reducible in that there exists modern techniques to efficiently reduce the emission sources, such as scrubbers, nitrogen oxides (NO_X) reduction catalysts, particle filters and electrification of vehicles.[32] Further efforts from the ambient air pollution regulation side are in great need to help reduce the associated mental healthcare burden in older adults.

© 2023 Qiu X et al. *JAMA Network Open.*

References

1. Climatology Lab; 2021. www.climatologylab.org/gridmet.html.
2. Abatzoglou JT. Development of gridded surface meteorological data for ecological applications and modelling. *International Journal of Climatology.* 2013;33(1):121-131.
3. NASA's MODIS satellite. https://ladsweb.modaps.eosdis.nasa.gov/.
4. https://www.nationalgeographic.org/maps/united-states-regions/.
5. Center for International Earth Science Information Network - CIESIN - Columbia University. Gridded Population of the World, Version 4 (GPWv4): Population Density, Revision 11. Palisades, New York: NASA Socioeconomic Data and Applications Center (SEDAC); 2018.
6. Summary File 3 Dataset. 2002. https://www.census.gov/data/datasets/2000/dec/summary-file-3.html (2002).
7. Summary File 1 Dataset. US Census 2011. https://www.census.gov/data/datasets/2010/dec/summary-file-1.html.
8. American Community Survey 1-Year Data (2005–2019). US Census; 2020. https://www.census.gov/data/developers/data-sets/acs-1year.html
9. Behavioral Risk Factor Surveillance System. 2020. https://www.cdc.gov/brfss/annual_data/annual_data.htm.
10. Dartmouth Health Atlas data. https://data.dartmouthatlas.org/.
11. Environmental Systems Research Institute (ESRI) hospital file. https://www.esri.com/en-us/industries/health/focus-areas/access-to-care.
12. Public Health Disparities Geocoding Project. https://www.hsph.harvard.edu/thegeocodingproject/covid-19-resources/.
13. National Ambient Air Quality Standards. 2022; https://www.epa.gov/criteria-air-pollutants/naaqs-table.
14. *Global Air Quality Guidelines - Update 2021.* Copenhagen, Denmark: WHO Regional Office for Europe: World Health Organization;2021.
15. Gold SM, Köhler-Forsberg O, Moss-Morris R, et al. Comorbid depression in medical diseases. *Nature reviews Disease primers.* 2020;6(1):69.
16. Huang CQ, Dong BR, Lu ZC, Yue JR, Liu QX. Chronic diseases and risk for depression in old age: a meta-analysis of published literature. *Ageing research reviews.* 2010;9(2):131-141.
17. Hernan M, Robins J. Causal Inference: What if. Boca Raton: Chapman & Hill/CRC. 2020.
18. Schisterman EF, Cole SR, Platt RW. Overadjustment bias and unnecessary adjustment in epidemiologic studies. *Epidemiology (Cambridge, Mass).* 2009;20(4):488.
19. Patten SB. Long-term medical conditions and major depression in the Canadian population. *The Canadian Journal of Psychiatry.* 1999;44(2):151-157.
20. Hernán MA, Hernández-Díaz S, Robins JM. A structural approach to selection bias. *Epidemiology (Cambridge, Mass).* 2004:615-625.
21. VanderWeele TJ, Ding P. Sensitivity Analysis in Observational Research: Introducing the E-Value. *Annals of internal medicine.* 2017;167(4):268-274.
22. Li H, Zhang S, Qian ZM, et al. Short-term effects of air pollution on cause-specific mental disorders in three subtropical Chinese cities. *Environmental research.* 2020;191:110214.
23. Thilakaratne RA, Malig BJ, Basu R. Examining the relationship between ambient carbon monoxide, nitrogen dioxide, and mental health-related emergency department visits in California, USA. *Science of the Total Environment.* 2020;746:140915.
24. Li D, Ji S, Guo Y, Sang N. Ambient NO2 exposure sex-specifically impairs myelin and contributes to anxiety and depression-like behaviors of C57BL/6J mice. *Journal of Hazardous Materials.* 2021;416:125836.
25. Qiu X, Danesh-Yazdi M, Wei Y, et al. Associations of short-term exposure to air pollution and increased ambient temperature with psychiatric hospital admissions in older adults in the USA: a case-crossover study. *The Lancet Planetary health.* 2022;6(4):e331-e341.
26. Kamo Y. Racial and ethnic differences in extended family households. *Sociological Perspectives.* 2000;43(2):211-229.
27. Lofquist D. Multigenerational households. Paper presented at: Annual Meeting of the American Sociological Association, Working Paper2013.
28. Dominici F, Peng RD, Barr CD, Bell ML. Protecting human health from air pollution: shifting from a single-pollutant to a multipollutant approach. *Epidemiology (Cambridge, Mass).* 2010;21(2):187-194.
29. Shi L, Wu X, Danesh Yazdi M, et al. Long-term effects of PM(2·5) on neurological disorders in the American Medicare population: a longitudinal cohort study. *The Lancet Planetary health.* 2020;4(12):e557-e565.

© 2023 Qiu X et al. *JAMA Network Open.*

30. Wang Y, Shi L, Lee M, et al. Long-term Exposure to PM2.5 and Mortality Among Older Adults in the Southeastern US. *Epidemiology (Cambridge, Mass)*. 2017;28(2):207-214.
31. Heusinkveld HJ, Wahle T, Campbell A, et al. Neurodegenerative and neurological disorders by small inhaled particles. *Neurotoxicology.* 2016;56:94-106.
32. Cooper CD, Alley FC. *Air pollution control: A design approach.* Waveland press; 2010.

eTable 1. ICD diagnosis codes for depression condition in the Medicare CCW (Reference period: 1-year, revised 02/2021)

Codes	Depression
**ICD-9**	DX 296.20, 296.21, 296.22, 296.23, 296.24, 296.25, 296.26, 296.30, 296.31, 296.32, 296.33, 296.34, 296.35, 296.36, 296.51, 296.52, 296.53, 296.54, 296.55, 296.56, 296.60, 296.61, 296.62, 296.63, 296.64, 296.65, 296.66, 296.89, 298.0, 300.4, 309.1, 311 (any DX on the claim)
**ICD-10** [1]	DX F31.30, F31.31, F31.32, F31.4, F31.5, F31.60, F31.61, F31.62, F31.63, F31.64, F31.75, F31.76, F31.77, F31.78, F31.81, F32.0, F32.1, F32.2, F32.3, F32.4, F32.5, F32.9, F33.0, F33.1, F33.2, F33.3, F33.40, F33.41, F33.42, F33.8, F33.9, F34.1, F43.21, F43.23 (any DX on the claim)
**Number/Type of Claims to Qualify** [2]	At least 1 inpatient, SNF, HHA, HOP, or Carrier claim with DX codes

[1] ICD-10 codes are effective 10/2015; effective dates for ICD-9 codes vary, but are valid through 09/2015. Researchers may be interested in confirming the code(s) of interest in the accompanying claims data files. [2] SNF refers to skilled nursing facility; HHA refers to home health agency; HOP refers to hospital outpatient. Carrier claims refer to claim types 71 and 72 (not durable medical equipment [DME] claim types 81 or 82), and excludes any claims for which line item Berenson-Eggers Type of Service (BETOS) code variable equals D1A, D1B, D1C, D1D, D1E, D1F, D1G (which is DME), or O1A (which is ambulance services). The intent of the algorithm is to exclude claims where the services do not require a licensed health care professional. When two claims are required, they must occur at least one day apart. DX denotes diagnosis.

© 2023 Qiu X et al. *JAMA Network Open.*

eTable 2. Lag-specific estimates for the associations between exposure to air pollutants and risk of late-life depression diagnosis in the Medicare over current (lag0) and past 5 years (lag1 – lag5) exposure window.

	**$PM_{2.5}$**		**NO_2**		**O_3**	
**Lag**	**HR (95% CI)** [a]	***P*** [b]	**HR (95% CI)**	***P***	**HR (95% CI)**	***P***
Lag0	1.027 (1.019-1.036)	<0.001	1.004 (1.001-1.006)	0.011	1.016 (1.011-1.020)	<0.001
Lag1	1.020 (1.012-1.028)	<0.001	1.003 (1.000-1.006)	0.026	1.014 (1.010-1.018)	<0.001
Lag2	1.010 (1.002-1.017)	0.014	1.003 (1.001-1.006)	0.011	1.013 (1.009-1.017)	<0.001
Lag3	1.001 (0.994-1.008)	0.807	1.005 (1.003-1.008)	<0.001	1.016 (1.012-1.020)	<0.001
Lag4	1.002 (0.995-1.009)	0.596	1.006 (1.004-1.009)	<0.001	1.018 (1.014-1.022)	<0.001
Lag5	0.996 (0.989-1.003)	0.266	1.007 (1.005-1.009)	<0.001	1.023 (1.018-1.027)	<0.001

[a] hazard ratio per 5 units increase in lag-specific annual average exposure adjusting for selected covariates; [b] z test p value.

© 2023 Qiu X et al. *JAMA Network Open.*

eTable 3. E-values at risk ratio scale for the main estimates.

	$PM_{2.5}$			NO_2			O_3		
**Measure**	**HR [a]**	**Lower [b]**	**Upper [c]**	**HR**	**Lower**	**Upper**	**HR**	**Lower**	**Upper**
Main Effect Estimate	1.009	1.000	1.018	1.006	1.003	1.009	1.021	1.016	1.026
E-value [d] (rare outcome)	1.10	1.01	-	1.08	1.06	-	1.17	1.15	-
E-value (common outcome)	1.09	1.01	-	1.07	1.05	-	1.14	1.12	-

[a] hazard ratio per 5 units increase in long-term average exposures (moving average of current and past 5 years of exposure) adjusting for selected covariates; [b] lower bound of 95% confidence interval; [c] upper bound of 95% confidence interval; [d] E-value defined as the minimum strength of association on the risk ratio scale that an unmeasured confounder would need to have with both the exposure and the outcome to fully explain away a specific exposure-outcome association, conditional on the measured covariates. (https://cran.r- project.org/web/packages/EValue/index.html; VanderWeele, T. J., & Ding, P. (2017). Sensitivity analysis in observational research: introducing the E-value. Annals of internal medicine, 167(4), 268-274.). Calculated with reference to online calculator: https://www.evalue-calculator.com/evalue/.

© 2023 Qiu X et al. *JAMA Network Open.*

eTable 4. Hazard ratio (HR) of depression diagnosis risk and 95% confidence intervals (CI) among the Medicare participants over 2005-2016 per 5 units increase in long-term average pollutant exposure (pollutant crude model results)

**Pollutant**	**HR (95% CI)** [a]
Single $PM_{2.5}$	1.013 (1.007-1.019)
Single NO_2	0.994 (0.992-0.996)
Single O_3	1.026 (1.021-1.030)
**Tri-pollutant**	
$PM_{2.5}$	1.025 (1.019-1.032)
NO_2	0.993 (0.991-0.995)
O_3	1.024 (1.020-1.029)

[a] hazard ratio per 5 units increase in long-term average exposures (moving average of current and past 5 years of exposure) without additional adjustment of other covariates.

© 2023 Qiu X et al. *JAMA Network Open.*

eTable 5. Annual average (standard deviation, SD) residential exposure to air pollutants among participants with comorbidities vs. without over study period.

	$PM_{2.5}$				NO_2				O_3			
	With Condition		Without Condition		With Condition		Without Condition		With Condition		Without Condition	
	average	SD	average	SD	average	SD	average	SD	average	SD	average	SD
**Cancer**	8.8	2.4	8.8	2.4	16.8	8.8	16.3	8.6	38.9	3.8	39.0	3.8
**Diabetes**	9.0	2.4	8.7	2.5	17.2	9.1	15.9	8.3	38.9	3.7	39.1	3.8
**COPD**	9.0	2.5	8.8	2.4	16.4	8.8	16.4	8.6	39.1	3.7	39.0	3.8
**Stroke**	9.1	2.5	8.8	2.4	16.8	8.8	16.3	8.6	39.0	3.8	39.0	3.8
**Alzheimer disease**	9.4	2.5	8.8	2.4	17.5	9.2	16.3	8.6	38.8	3.8	39.0	3.8
**Dementia**	9.3	2.5	8.7	2.4	17.2	9.1	16.3	8.5	38.9	3.8	39.0	3.8
**Hypertension**	8.9	2.4	8.4	2.5	16.5	8.6	16.0	8.5	39.0	3.8	39.1	4.1
**CHF**	9.1	2.5	8.7	2.4	17.2	9.2	16.1	8.4	38.9	3.8	39.0	3.8

© 2023 Qiu X et al. *JAMA Network Open.*

eFigure 1. Directed acyclic graph (DAG) causal diagram showing the pathways from air pollution exposure to late-life depression and their relationships with comorbidities.

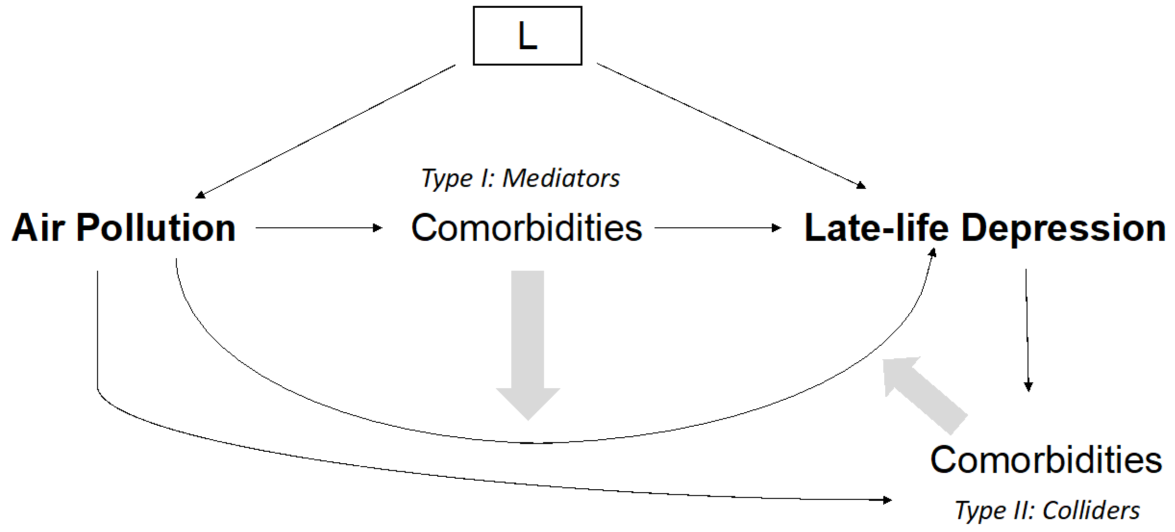

Here, an arrow tail that starts from a variable (A) and ends on a second variable (B) means that A causes B. A series of adjacent arrows in the graph, regardless of direction, forms a path. L represents a set of key confounders and putting a box surrounding it means adjusting for them. There are two types of comorbidities (physical and mental) here, type I are potential mediators for the air pollution – late-life depression associations while type II are potential colliders for the associations.

© 2023 Qiu X et al. *JAMA Network Open.*

eFigure 2. Spatial distribution maps of air pollutants studied. The three panels present the average concentrations of a) annual average $PM_{2.5}$ ($\mu g/m^3$), b) NO_2 (ppb), and c) O_3 (ppb) at zip code level across the contiguous United States in 2010, respectively.

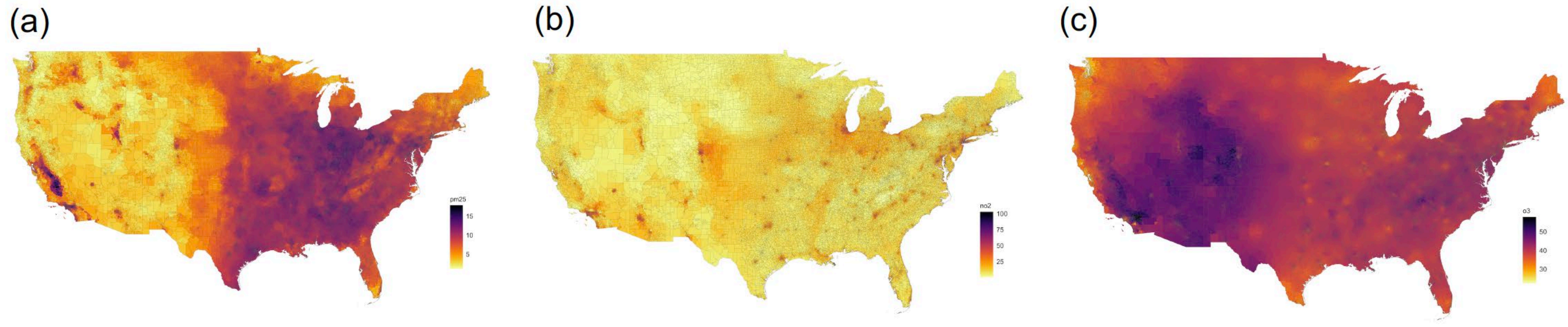

© 2023 Qiu X et al. *JAMA Network Open.*

eFigure 3. Sub-group results by individual and neighborhood factors.

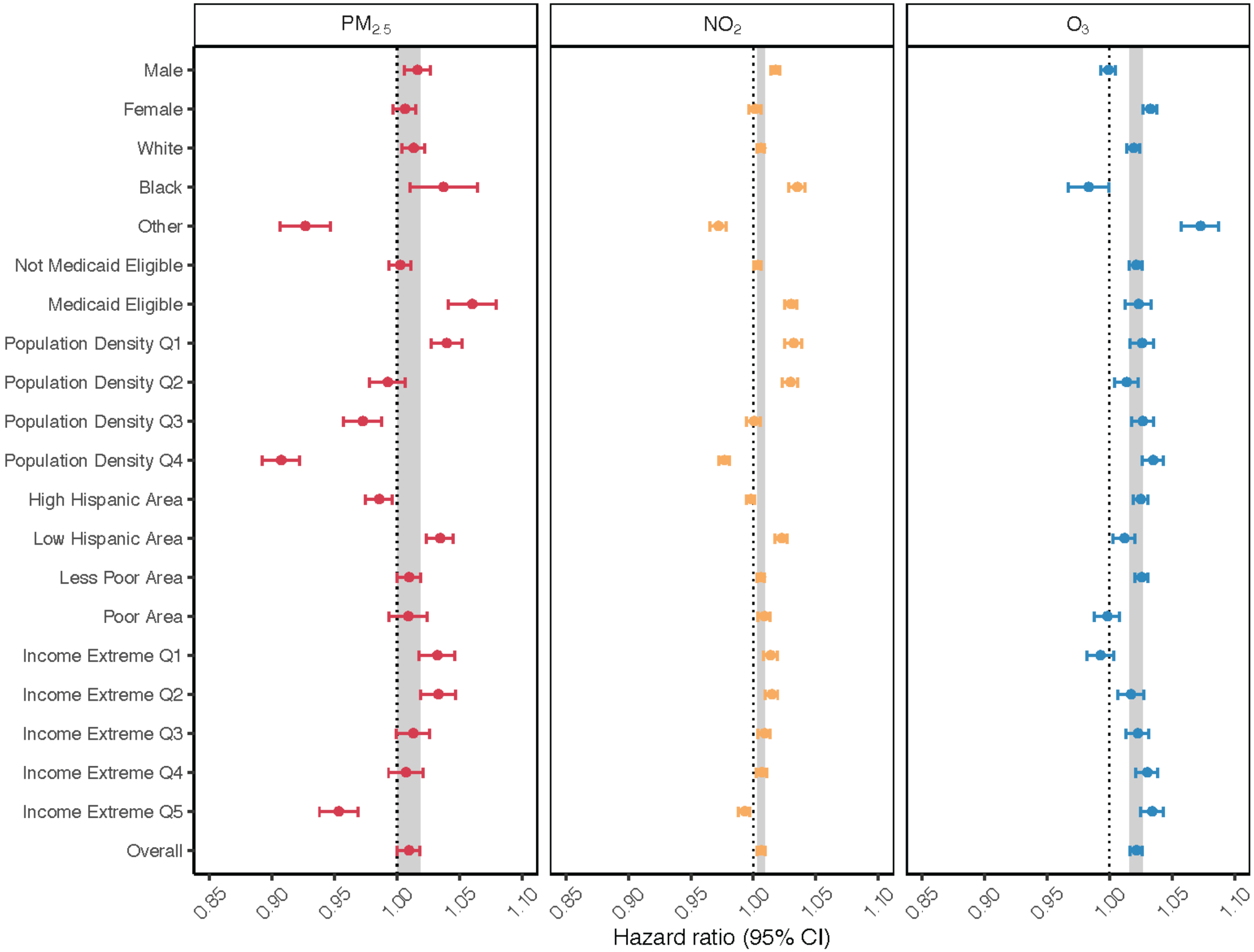

The plots show percentage (%) change of depression diagnosis risk and 95% confidence intervals per 5 units increase in long-term average pollutants (PM2.5 (µg/m3), NO2 (ppb), and O3 (ppb)) adjusting for covariates. To assess potential effect modification (EM) by individual and neighborhood factors, we included multiplicative interaction terms between potential modifiers and each of the

© 2023 Qiu X et al. *JAMA Network Open.*

three pollutants. We examined potential interaction by sex (Males vs. Females), race groups (White vs. Black vs. Other races: including Asian, Pacific Islander, Native American & multiracial), individual Medicaid eligibility (Yes vs. No), population density (divided by quartiles, Q1 - Q4, low to high), area percent of Hispanic population (High vs. Low, divided by median percentile), area percent of individuals in poverty (Poor vs. Less poor using cutoff of over 20% residents living in poverty in the area) and area income extreme index (ICE, divided by quintiles Q1 - Q5, low to high).

© 2023 Qiu X et al. *JAMA Network Open.*

eFigure 4. Distributions of residential ambient air pollutants level differences post - pre moving into a new address among participants with comorbidities over the study period.

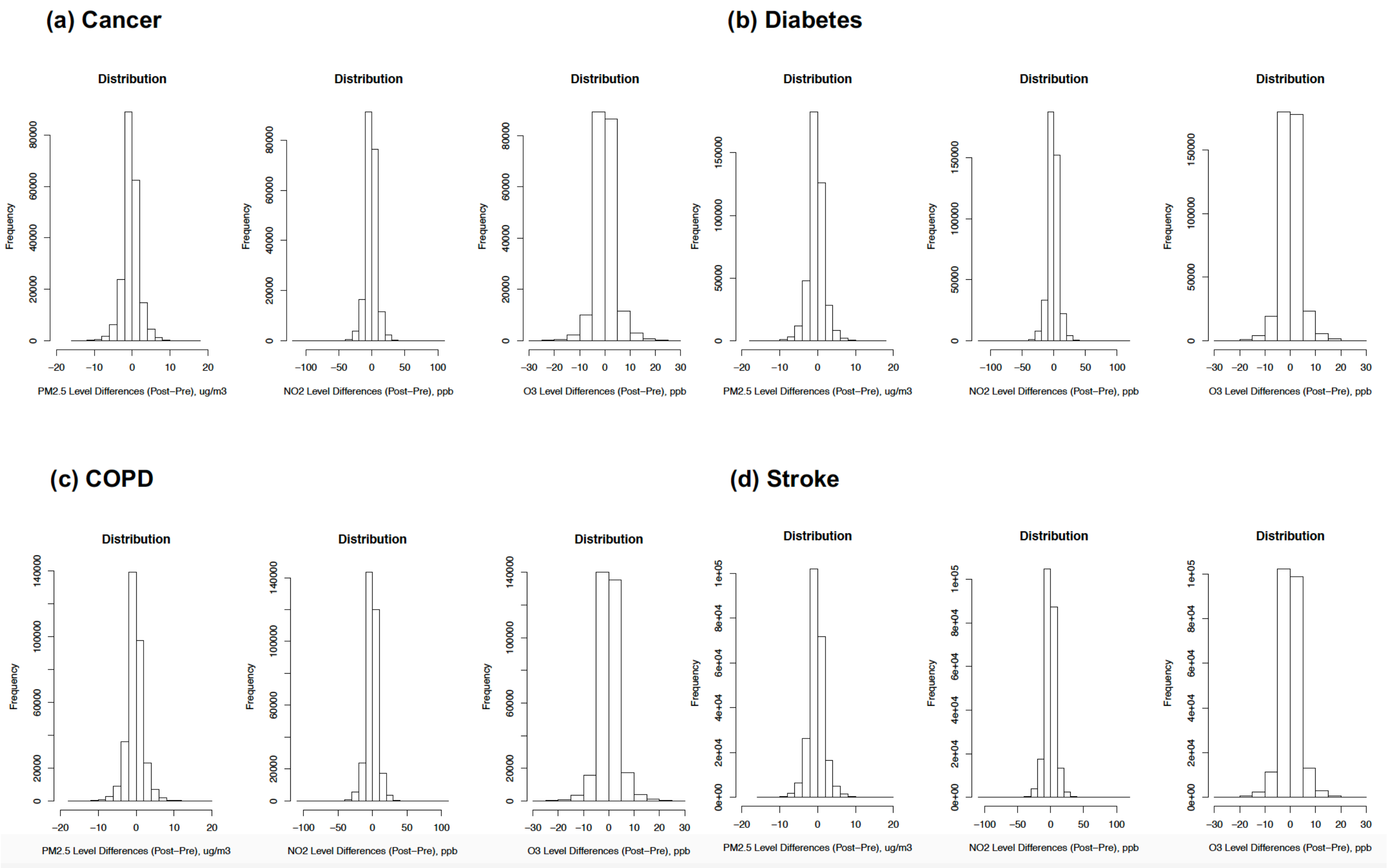

© 2023 Qiu X et al. *JAMA Network Open.*

eFigure 4. Distributions of residential ambient air pollutants level differences post - pre moving into a new address among participants with comorbidities over the study period. (Continued)

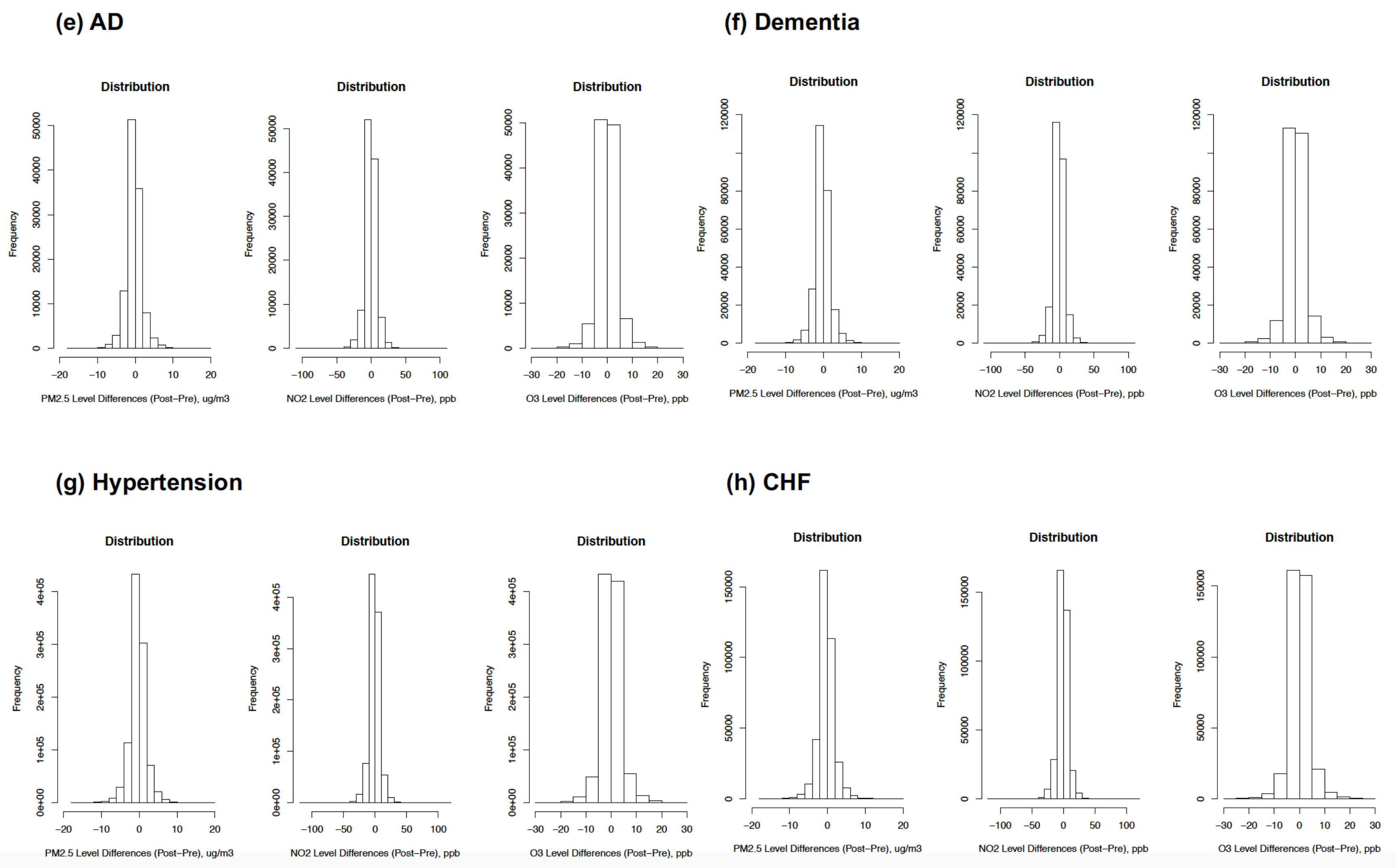

© 2023 Qiu X et al. *JAMA Network Open.*
