# Supplementary material for: Association of Long-term Exposure to Air Pollution With Late-Life Depression in Older Adults in the US
Source: JAMA Netw Open. 2023 Feb 10;6(2):e2253668. doi: 10.1001/jamanetworkopen.2022.53668 (PMC9918878; doi:10.1001/jamanetworkopen.2022.53668)
Supplement: Supplement 2. — Data Sharing Statement [file jamanetwopen-e2253668-s002.pdf]

Data Sharing Statement

Qiu. Association of Long-term Exposure to Air Pollution With Late-Life Depression in Older Adults in the US. *JAMA Netw Open*. Published February 10, 2023.
doi:10.1001/jamanetworkopen.2022.53668

Data
Data available: No

Additional Information
Explanation for why data not available: The rules governing the Medicare dataset prohibit any sharing of the health datasets being used for our epidemiologic research. Restricted by our Data Use Agreement with the U.S. Centers for Medicare & Medicaid Services, the Medicare data that support the findings of this study are neither sharable nor publicly available from us. Academic and non-profit researchers who are interested in using Medicare data should contact the US Centers for Medicare & Medicaid Services directly to obtain their own datasets upon completion of a Data Use Agreement.
